# A comprehensive landscape of the *Gossypium arboreum* circRNAome under multiple abiotic stresses

**DOI:** 10.3389/fpls.2026.1791897

**Published:** 2026-03-10

**Authors:** Liangdan Fei, Yifan Zhou, Tao Huang, Kun Wang

**Affiliations:** College of Life Sciences, Wuhan University, Wuhan, China

**Keywords:** abiotic stress, ceRNA network, chloroplast, circRNA, *Gossypium arboreum*, PsbA

## Abstract

Circular RNAs (circRNAs) serve as key post-transcriptional regulators in plant stress adaptation. Here, we comprehensively characterize the circRNA landscape of cotton (*Gossypium arboreum*) under multiple abiotic stresses condition using RNase R-enhanced sequencing. Through a stringent KNIFE-based algorithm pipeline, we identified 4,365 high-confidence circRNAs. Mechanistically, circRNA biogenesis was associated with long flanking introns and exhibited complex patterns of alternative splicing, revealing conserved production propensities but dynamic splice sites across species. Nuclear circRNAs frequently exhibited expression patterns decoupled from their host genes, typically in a stress-specific manner. By integrating miRNA-seq data, we constructed a circRNA-miRNA-mRNA regulatory network and found it centered mainly on cotton-specific miRNAs. Notably, we discovered an extraordinary dominance of chloroplast-derived circRNAs, accounting for over 80% of the total circRNAs repertoire. These chloroplast circRNAs clustered dominantly at clustering at the 3’ terminus of the photosynthetic gene *psbA* gene, suggesting a specialized post-transcriptional regulatory mechanism within the chloroplast. This study provides a high-resolution cotton circRNA atlas and highlights *psbA*-derived circRNAs as potential molecular targets for improving environmental resilience in crop.

## 1 Introduction

Cotton is a fundamental industrial raw material for the global textile industry and a strategic resource vital to China’s national economy. As the world’s premier natural fiber crop, it accounts for approximately 20% of global fiber production and supports a multi-billions dollar value chain (Textile Exchange, 2024; OECD/FAO, 2024). However, the developmental period from flowering to boll opening is protracted, rendering the crop highly susceptible to drought and high temperatures, which can lead to a significant reduction in both yield and fiber strength (Sarwar et al., 2018; Zafar et al., 2023). Xinjiang, China’s primary cotton growing region in China, faces unique climatic challenges. Despite historical “warm-wetting” observations, recent evidence identifies a critical wet-to-dry shift (Yao et al., 2021) and intensifying hydroclimatic instability driven by extreme evaporation (Wang et al., 2025), posing persistent threats to the region’s agricultural resilience (Xu et al., 2025). This escalating climatic risks has been identified as a primary driver of yield fluctuations, severely constraining both cotton yield and fiber quality (Li et al., 2020). These multifaceted abiotic stressors—including heat, cold, salt, and UV radiation—pose significant threats to sustainable cotton production (Patil et al., 2024). Therefore, a comprehensive understanding of the molecular mechanisms underlying cotton stress resistance is crucial for the genetic improvement of climate-resilient and high-yield cultivars capable of withstanding these complex environmental challenges.

The emergence of non-coding RNAs (ncRNAs) offers a novel perspective for dissecting the mechanisms underlying crop stress resistance. In *G. arboreum*, previous systematic identification and high-resolution annotation of mRNAs and long non-coding RNAs (lncRNAs) have unveiled the complex regulatory landscapes governing cotton development and environmental adaptation (Wang et al., 2019a; Zheng et al., 2020). These datasets provide a robust foundation for exploring deeper regulatory layers, such as circular RNAs (circRNAs). Characterized by their covalently closed continuous loops lacking 5’ caps and 3’ poly(A) tails, circRNAs represent a widespread and diverse class of eukaryotic ncRNAs with essential roles in cellular homeostasis (Chen, 2016). Beyond their structural stability, circRNAs play pivotal roles in diverse biological processes and gene expression regulation, suggesting they are functional components of the transcriptome rather than mere splicing by-products (Hansen et al., 2013; Jeck et al., 2013).

Over the past decade, plant circRNA research has expanded substantially. For instance, overexpression of CircR5g05160 enhances rice resistance to fungal pathogens, while grape-derived Vv-circATS1 improves cold tolerance in Arabidopsis thaliana (Fan et al., 2020; Gao et al., 2019). Beyond their roles in environmental adaptation, circRNAs can exert sophisticated regulatory effects directly on their host loci. A prominent recent study demonstrated that circRNAs derived from miR156d can promote rice heading by repressing the transcription elongation of pri-miR156d through the formation of R-loops (Su et al., 2025). These findings highlight that circRNAs modulate plant development and stress responses through diverse and evolutionarily conserved mechanisms. Nevertheless, a comprehensive landscape of the cotton circRNAome—including its expression and interaction patterns under diverse abiotic stresses—remains largely elusive. To bridge this gap, we employed G. arboreum to systematically dissect circRNA biogenesis, stress specificity expression, and the intricate circRNA–miRNA regulatory circuitry. By integrating RNase R-treated RNA-seq with KNIFE-based algorithm identification pipelines, we generated detailed circRNA expression atlases under cold, salt, heat, and UV-B treatments. This work provides novel molecular modules and fundamental insights for improving multi-stress tolerance in cotton.

## 2 Materials and methods

### 2.1 Plant growth and treatments

The plant material utilized in this research was the diploid AA-genome *Gossypium arboreum* cv. Shixiya-1 (SXY-1). These plants were cultivated in an automated greenhouse maintained under the following controlled conditions: 28 °C, a 16 h light/8 h dark photoperiod, and 65% relative humidity. Two-week-old cotton seedlings were subjected to four abiotic stress treatments (Makarevitch et al., 2015). Stress conditions included heat (50 °C, 5 h) and cold (5 °C, 15 h) to trigger thermal-responsive regulators (Ni et al., 2021; Li et al., 2024), salt (irrigated with a 500 mM NaCl solution) to simulate severe salinity-induced ionic and osmotic stress (Zhang et al., 2017), and UV-B (1.24 μmol m^−2^ s^−1^, 2 h) to investigate rapid defense signaling (Song et al., 2024).

### 2.2 RNA extraction and circRNA sequencing

High-quality total RNA was used for circRNA library construction. To enrich for circular transcripts, total RNA was digested with RNase R to remove linear RNA. The resulting circRNA was purified using Agencourt RNA Clean XP magnetic beads. The purified circRNA was then fragmented and used as a template for cDNA synthesis. The cDNA fragments underwent end repair, “A”-tailing, and adaptor ligation, followed by final PCR amplification. Library quality was assessed using an Agilent 2100 Bioanalyzer for size distribution and quantified via qPCR. Qualified libraries were subsequently sequenced using PE100 high-throughput sequencing on the Illumina HiSeq 2500 platform.

### 2.3 CircRNA identification

Following acquisition of the sequencing data, quality control was performed using fastp v0.20.1 (Chen et al., 2018b). Subsequently, the clean reads were aligned to the *G. arboreum* SXY-1 genome (CRI assembly) (Du et al., 2018) with IGIA annotations (Wang et al., 2019a) using STAR v2.7.2b (Dobin et al., 2012). To identify chloroplast-derived circRNAs, we utilized the *G. arboreum* chloroplast genome sequence (NC_016712.1) as the reference (Wu et al., 2018).

This alignment facilitated the identification of all linear and back-splicing reads. circRNAs were identified in each sample using the KNIFE v1.4 software (https://github.com/lindaszabo/KNIFE), which also assigned a reliability score to each candidate. Finally, after excluding artifacts originating from ultra-long distance circularization, the bona fide circRNAs were retained based on stringent score cutoffs. Specifically, circRNAs with a score greater than 0.1 were retained from our RNase R-treated samples. For the non-stress control group, we utilized previously published high-quality RNA-seq datasets (Wang et al., 2019a), and retained circRNAs with a score greater than 0.9 to ensure high confidence. This integrated approach allowed for a robust comparison between RNase R-enriched libraries and standard transcriptomic data.

### 2.4 CircRNA validation

The existence of candidate circRNAs was validated by PCR using divergent primers specifically designed to amplify the back-splice junctions (BSJs). To compensate for the low abundance of circRNAs, PCR was performed for 40 cycles using an increased amount of cDNA template. The amplified product of the expected size was purified and subjected to Sanger sequencing. The resulting sequence was aligned to the circRNA sequence to confirm the precise location and presence of the BSJ.

#### 2.4.1 Quantification and differential expression analysis of circRNAs

To quantify expression levels, the alignment results for all samples were sorted using SAMtools v1.9 (Li et al., 2009). Subsequently, StringTie v2.0.6 (Pertea et al., 2015) was employed to calculate the expression levels for all genes and circRNAs. Specifically, expression values from RNase R-treated samples represented circRNA levels, while those from control samples represented linear transcript levels. Differential expression analysis between samples was performed using the edgeR v3.32.1 package in R v4.0.2 (Robinson et al., 2009).

#### 2.4.2 miRNA-related analysis and target prediction

To investigate the expression profiles of miRNAs under the applied abiotic stresses, small RNA sequencing (sRNA-seq) was performed on all collected samples. Following quality control and adapter trimming, the resulting sequencing reads were aligned to the *G. arboreum* genome. The identification and annotation of known and novel miRNAs were conducted using the sRNAanno annotation pipeline sRNAminer v1.0.0 (Chen et al., 2021). Based on these annotations, the expression levels of each miRNA in every sample were calculated. Potential miRNA-circRNA and miRNA-gene interactions were predicted using three independent computational tools: miRanda v3.3a (Enright et al., 2003), the TargetFinder software (Fahlgren and Carrington, 2010), and the psRNATarget online tool (Dai and Zhao, 2011). Only those interactions consistently identified and predicted by all three software platforms and met the established thermodynamic criteria were retained as highly reliable results for the construction of the circRNA-miRNA-mRNA regulatory network and downstream functional analysis.

## 3 Results

### 3.1 Identification of stress-responsive circRNAs in *G. arboreum*

We performed RNase R-treated RNA sequencing (circRNA-seq) on two-week-old *G. arboreum* seedlings subjected to four distinct abiotic stresses to profile their circular RNAs (circRNAs) (**Supplementary Table 1**). First, the sequencing quality was evaluated by analyzing the genomic distribution of clean reads, with the majority of clean reads aligning to genic regions (**Supplementary Figure 1A**). Furthermore, high correlation coefficients between biological replicates across all treatments, confirming the high reproducibility of our data and their suitability for reliable circRNA identification (**Supplementary Figure 1B**).

**Figure 1:**
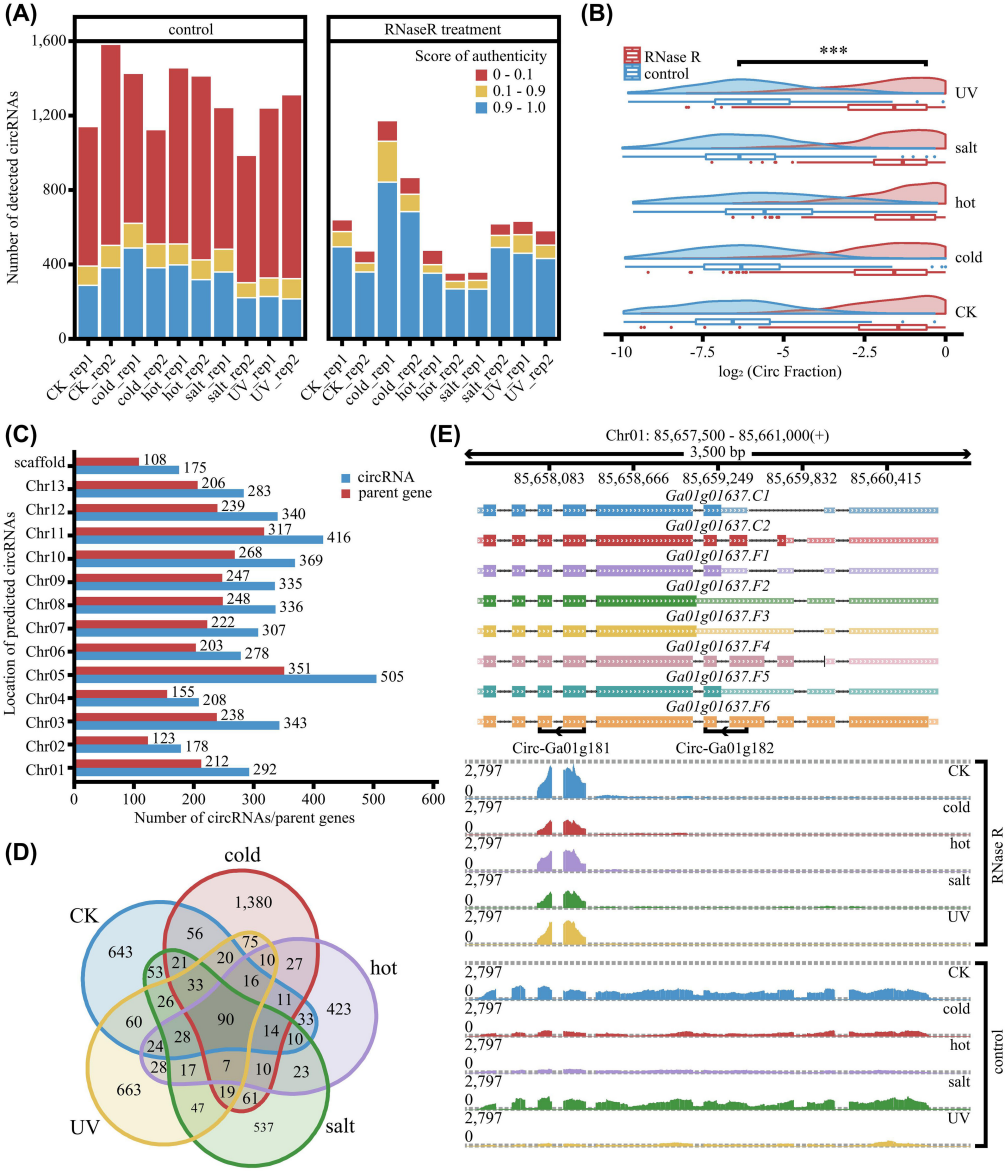
Identification of circRNAs in *G. arboreum*. **(A)** The numbers of detected distinct back-splicing events and their corresponding authenticity scores across different stress conditions. **(B)** Distribution of circular-to-linear ratios at BSJ sites detected in RNase R-treated samples versus controls. Statistical significance was determined using Fisher’s exact test. **(C)** Chromosomal distribution of identified circRNAs and their host genes across the 13 chromosomes. **(D)** Venn diagram illustrating the overlap of circRNAs identified across different conditions. **(E)** Read coverage and gene models for two representative circRNAs, Circ-Ga01g181 and Circ-Ga01g182. The scales were normalized by RPKM. The symbol *** indicates a statistically significant difference at the level of P < 0.001.

CircRNAs were predicted using the KNIFE pipeline, which assigned a reliability score to each candidate. The score distribution demonstrated that RNase R treatment effectively reduced low-scoring candidates and enriched those with high scores, thereby improving overall prediction fidelity (**Figure 1A**). Consistent with this, linear RNA abundance was markedly reduced post-digestion, validating the enzymatic enrichment (**Figure 1B**). However, false positives at BSJs remain an inherent challenge, as evidenced by the minimal overlap between digestion and non-digestion sets (**Supplementary Figure 1C**). To address this, we implemented a stringent filtering strategy to discriminate potential artifacts from bona fide circRNAs based on whether the BSJs are intragenic or intergenic. Analysis of the reliability score distribution showed that candidates with scores between 0–0.1 in RNase R-treated data, and 0–0.9 in control data, were predominantly supported by intergenic BSJs, indicating low credibility. Therefore, we applied dual thresholds: circRNAs with scores > 0.1 were retained from RNase R-treated samples, and those with scores > 0.9 from control samples. By integrating the results from both filtering pipelines, we identified 4,365 high-confidence unique circRNAs for downstream analysis (**Supplementary Table 2**). Notably, 2,591 of these candidates were supported by a single back-splice junction (BSJ) read in a single sample, indicating that the cotton circRNA landscape remains complex and has not yet reached a saturation plateau (**Supplementary Table 3**).

We further analyzed the chromosomal distribution of the identified circRNAs and their host genes. The majority were distributed across chromosomes 1 to 13, with only 175 mapped to unanchored scaffolds, supporting the notion that circRNA biogenesis is a genome-wide phenomenon in cotton (**Figure 1C**). Next, we examined their expression patterns under stress. Profiling revealed highly condition-specific expression: 1380, 423, 537, and 663 circRNAs were uniquely detected under cold, heat, salt, and UV-B conditions, respectively. By contrast, only 90 circRNAs were common to all four treatments (**Figure 1D**). Finally, to visually confirm the efficacy of our identification, we examined the read coverage at a representative locus (**Figure 1E**). In control samples, reads spanned the entire gene body, indicative of full-length linear transcripts. After RNase R treatment, these background reads were markedly depleted, whereas signals at the BSJs remained robust. This selective enrichment demonstrates that RNase R effectively removes linear RNA background, thereby ensuring the high fidelity of our circRNA identification.

### 3.2 Experimental validation of predicted circRNAs

To experimentally validate the identified circRNAs, we selected 33 high-confidence candidates based on their variable back-splicing read counts (**Supplementary Table 3**). Divergent primers targeting the BSJs were designed for each candidate. All candidates were successfully amplified by PCR, and subsequent Sanger sequencing unequivocally verified that the sequences matched the predicted junctions (**Supplementary Figure 2**).

**Figure 2:**
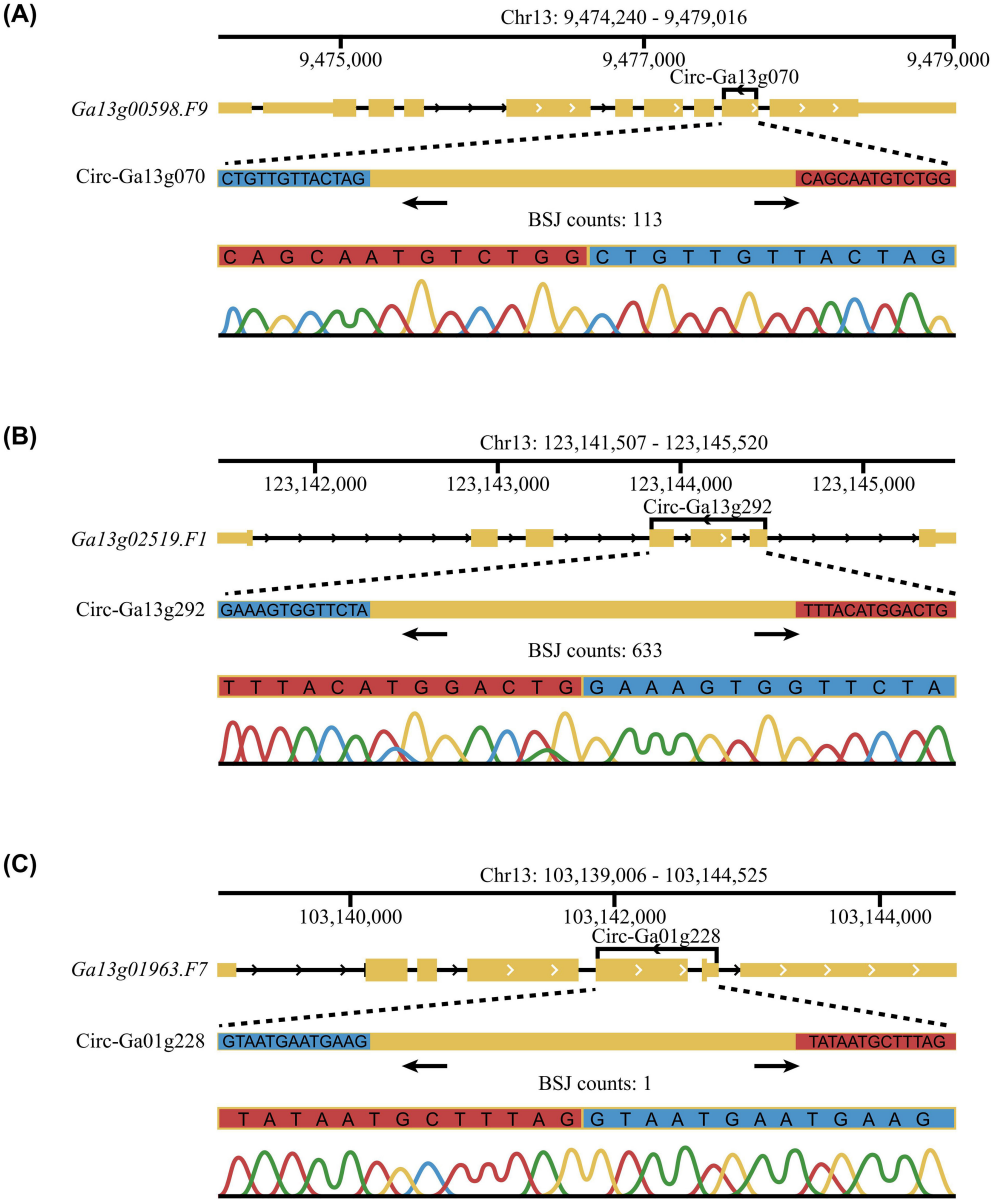
Experimental validation of identified circRNAs using RT-PCR and Sanger sequencing. **(A–C)** Genomic structures and PCR validation of three representative circRNAs: the single-exonic Circ-Ga13g070 **(A)**, the multi-exonic Circ-Ga13g292 **(B)**, and the multi-exonic Circ-Ga01g228 **(C)**.

This validation strategy successfully confirmed circRNAs across a spectrum of structural features and expression levels. For instance, Circ-Ga13g070, a single-exonic circRNA with high read support, was validated (**Figure 2A**). We also confirmed multi-exonic circRNAs, including Circ-Ga13g292 and Circ-Ga01g228, which represented high and very low read count candidates, respectively (**Figures 2B, C**). This comprehensive experimental confirmation underscores the sensitivity and reliability of our circRNA sequencing and annotation pipeline, affirming the genuine biological existence of these novel circRNAs in *G. arboreum*.

### 3.3 Characterization of circRNAs in *G. arboreum*

#### 3.3.1 Genomic features and biogenesis propensities of cotton circRNAs

**Figure 3:**
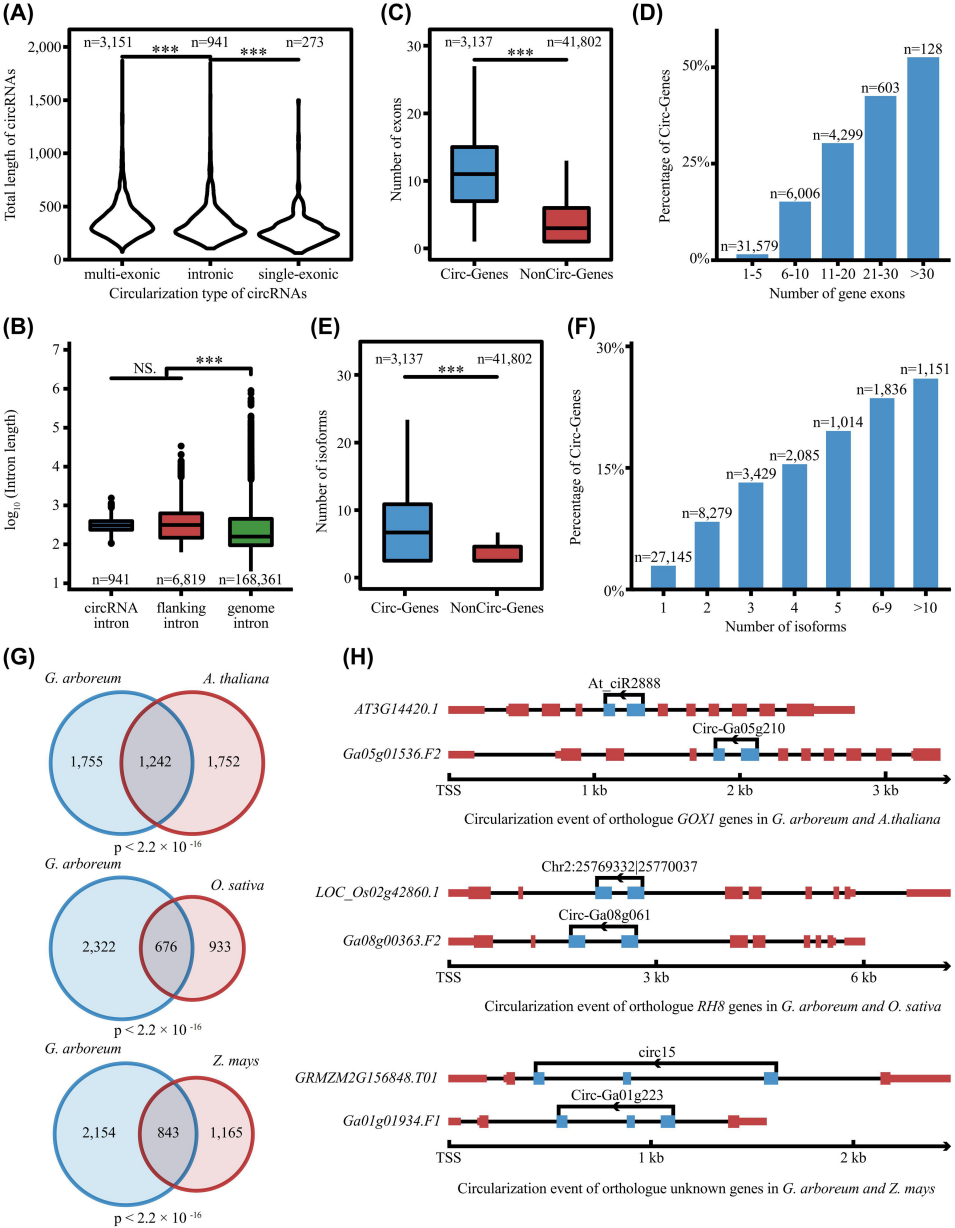
Genomic features and evolutionary conservation of cotton circRNAs. **(A)** Violin plot showing the length distribution of circRNAs classified by their genomic origin. **(B)** Comparison of intron lengths among circRNA-flanking introns, circRNAs introns, and genome-wide introns. **(C)** Boxplot comparing the number of exons in circRNA-host genes versus non-host genes. **(D)** Bar chart showing the proportion of genes producing circRNAs, categorized by their total exon number. **(E)** Boxplot comparing the number of isoforms in circRNA-host genes versus non-host genes. **(F)** Bar chart showing the proportion of genes producing circRNAs, categorized by their isoform count. In **(A–F)**, statistical significance was determined using Fisher’s exact test. **(G)** Venn diagram showing the overlap of circRNA host gene orthologs between *G. arboreum* and *A. thaliana*, *O. sativa*, and *Z. mays*. Significance of the overlap was measured by Hypergeometric test. **(H)** Schematic representation of conserved circularization events and splice sites in orthologous genes. The symbol *** indicates a statistically significant difference at the level of P < 0.001.

To systematically explore the molecular characteristics of cotton circRNAs, we began by analyzing their structures and lengths. The identified circRNAs were classified into three types: multi-exonic, single-exonic, and intronic formed by head-to-tail splicing of multiple exons, single exon and intron, respectively. Multi-exonic circRNAs were overwhelmingly dominant (3,151), followed by intronic (941) and single-exonic (273). Overall, circRNAs lengths peaked around 250 bp, with multi-exonic circRNAs being significantly longer than the other two types (**Figure 3A**).

Next, we compared the lengths of introns flanking circRNA-forming exons (flanking introns), introns contained within circRNAs (circRNA-introns), and average genomic introns. Both circRNA-associated intron categories were significantly longer than genomic introns, strongly supporting the notion that longer introns facilitate circRNAs biogenesis (**Figure 3B**).

We further compared the genomic features of circRNA-host genes versus non-host genes. CircRNA-host genes possessed significantly more exons (average 12) than non-host genes (average 4), and exon number was positively correlated with circRNA production propensity (**Figures 3C, D**). Also, circRNA-host genes produced more mRNA isoforms (average 3) than non-host genes (average 1) (**Figures 3E, F**).

In summary, circRNA formation in cotton is non-random and preferentially occurs in genes with longer introns and more exons—features intrinsically linked to a higher capacity for complex alternative splicing.

#### 3.3.2 Evolutionary Conservation and the Mechanism of Ring Formation

Although canonical models posit that circRNA biogenesis is driven by inverted complementary sequences (ICSs) or repetitive sequences in flanking introns, which promote proximity via base-pairing, our analysis in cotton revealed a distinct pattern (Xiao et al., 2020). We found no significant difference in the length or complementarity of ICSs flanking circRNAs compared to those flanking randomly selected linear exon (**Supplementary Figure 3A**). Moreover, repetitive sequence abundance was even lower in circRNA-flanking introns than in random intronic sequences (**Supplementary Figures 3B, C**). These results revealed that cotton circRNAs biogenesis was largely independent of these canonical cis-regulatory features, suggesting a greater potential reliance on other mechanisms.

To evaluate evolutionary conservation, we performed a comparative analysis using circRNA-seq data from *Arabidopsis thaliana* (Ye et al., 2015)*, Oryza sativa* (Wang et al., 2019b), and *Zea mays* (Chen et al., 2018a). The significant overlap of circRNA-producing orthologs confirms the conservation of circRNA biogenesis potential at the gene level (**Figure 3G**). We then investigated whether the precise BSJs themselves were conserved. By comparing circRNAs from orthologous genes, we classified them into three categories based on splice site conservation: “Accordant”, “Half-accordant”, and “Unaccordant”. The “Unaccordant” category predominated in all cross-species comparisons, indicating that specific circularization sites are highly dynamic. (**Supplementary Figure 3D**). Despite this widespread divergence, we identified several instances of positional conservation. This is exemplified by the *GOX1* and *RH8* orthologs, which produce circRNAs from identical genomic coordinates across the compared species (**Figure 3H**). Thus, while the propensity of a gene to produce circRNAs is conserved, the specific circularization sites are highly dynamic across species.

### 3.4 Analysis of circRNA and host gene expression under abiotic stress

**Figure 4:**
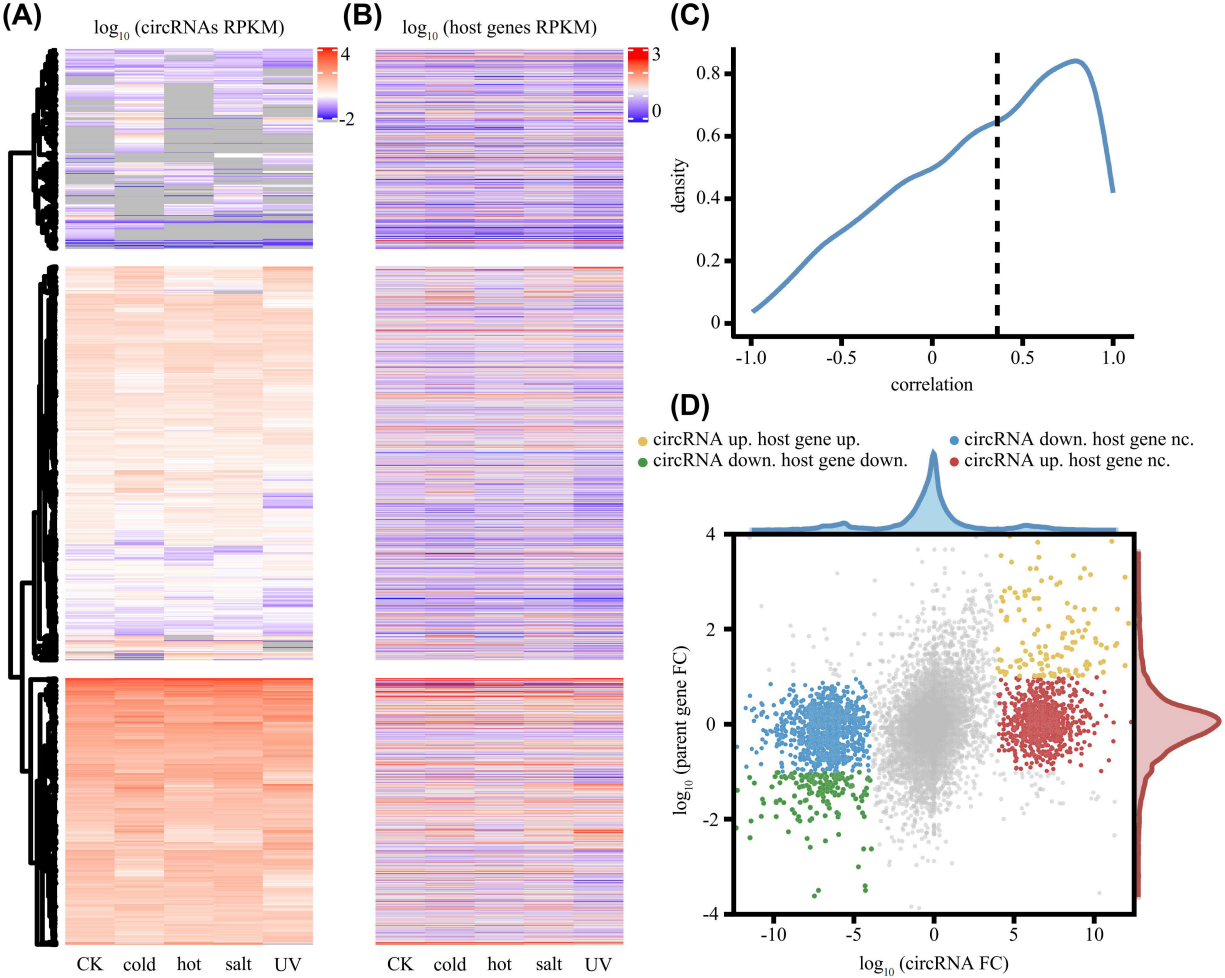
Expression patterns and correlation analysis of circRNAs and their host genes under abiotic stresses. **(A)** Hierarchical clustering heatmap showing the expression profiles of detected circRNAs across five conditions. **(B)** Heatmap displaying the expression levels of the corresponding host genes across the same samples. **(C)** Density plot illustrating the distribution of Pearson correlation coefficients calculated between the expression patterns of circRNAs and their cognate host genes. **(D)** Scatter plot comparing the expression fold changes (FC) of circRNAs and their host genes between control and conditions. The marginal density plots show the distribution of fold changes for each axis.

To understand how abiotic stress reshapes the circRNA landscape in *G. arboreum*, we compared the expression profiles of all identified circRNAs and their host genes across all samples. Heatmap visualization revealed distinct circRNA expression patterns, and differential expression analysis further quantified these stress-specific responses (**Figures 4A, B**). Notably, cold and UV-B stress provoked the strongest responses, characterized by a predominant upregulation (306 and 290 DE circRNAs, respectively). Salt stress, however, triggered a net downregulation (141 DE circRNAs), while heat stress elicited the weakest response (58 DE circRNAs). These results demonstrate that distinct abiotic stressors modulate the circRNA transcriptome in specific ways.

Beyond stress-specific expression, a key finding was the frequent decoupling of circRNA abundance from host gene transcription. Although a subset of circRNAs showed high expression correlation with their host genes (peak ~0.8), the overall median Pearson correlation coefficient was low (0.34) (**Figure 4C**). This regulatory independence was further underscored by scatter plot, which revealed that many circRNAs were differentially expressed even when their host gene transcripts remained stable (**Figure 4D**). Collectively, these data demonstrate that the accumulation of a substantial fraction of cotton circRNAs is governed by independent stress-specific regulatory mechanisms, rather than being passive byproducts of host gene transcription.

### 3.5 The potential regulatory role of circRNAs via the miRNA sponge mechanism in *G. arboreum*

**Figure 5:**
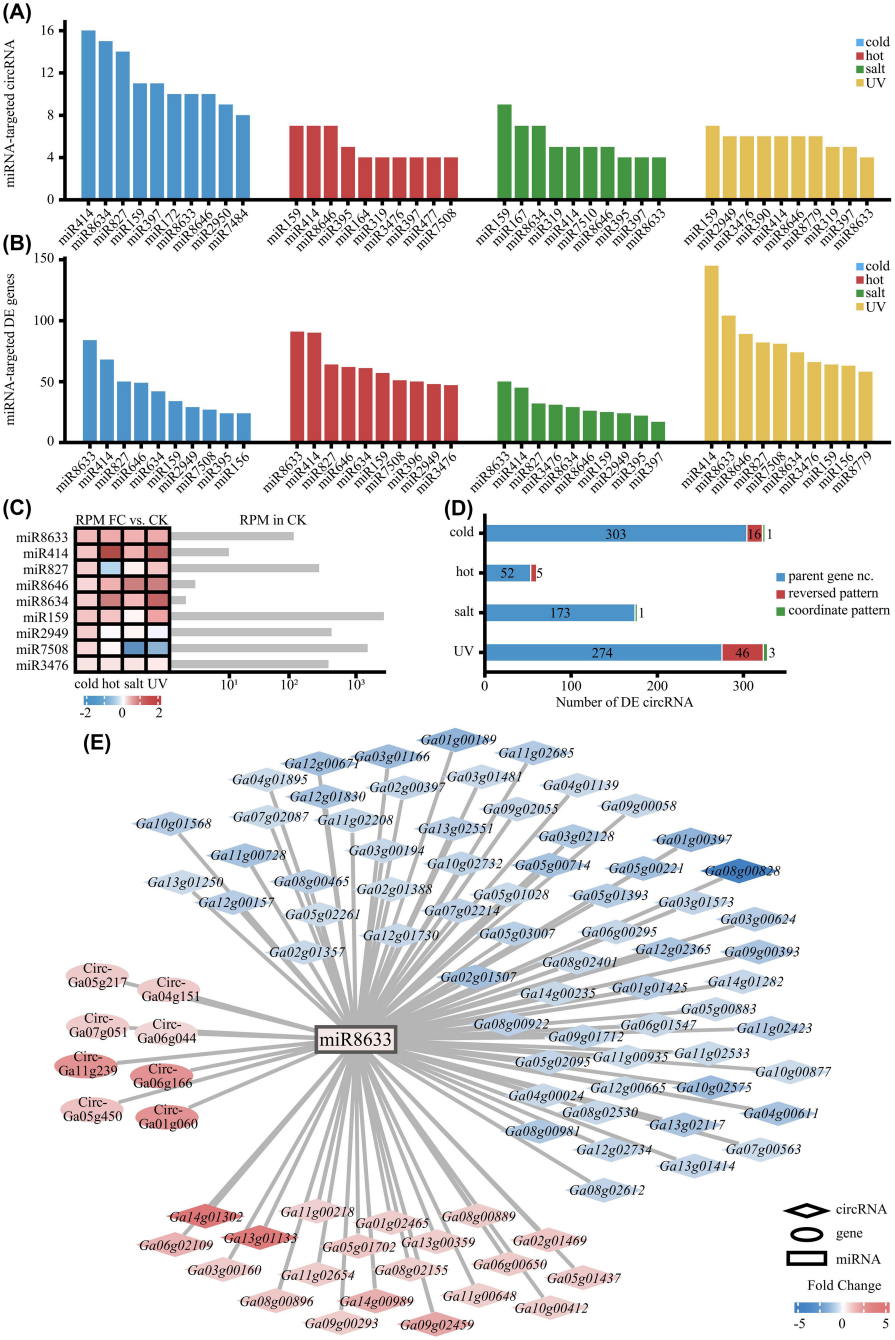
The circRNA-miRNA-mRNA network analysis. **(A)** Number of predicted target circRNAs for the top 10 miRNAs under each abiotic stress condition. **(B)** Number of predicted target differentially expressed genes for the top 10 miRNAs under each abiotic stress condition. **(C)** Expression patterns of key hub miRNAs. The heatmap shows the fold change in reads per million (RPM) of selected miRNAs under stress versus the control. The adjacent bar plot displays their basal expression levels in the CK condition. **(D)** Classification of DE circRNAs based on their expression patterns with their host genes. “Reversed pattern” indicates circRNAs acting as “mRNA up/circRNA down” or vice versa, while “Coordinated pattern” suggests potential co-expression. **(E)** A circRNA-miRNA-mRNA interaction network centered on miR8633 under cold stress. Lines represent predicted interactions. Node color and intensity represent the expression fold change of the corresponding RNA compared to CK.

CircRNAs have been widely reported to act as competitive endogenous RNAs, or miRNA sponges, sequestering miRNAs to modulate their target mRNAs (Hansen et al., 2013), which plays a pivotal role in fine-tuning plant stress responses (Yin et al., 2024). We therefore investigated this potential in *G. arboreum*. Bioinformatic predictions identified 656 circRNAs with potential miRNA-binding sites and 6,939 miRNA target mRNAs (**Supplementary Tables 4**–**5**). Then we performed small RNA sequencing (sRNA-seq) on samples subjected to cold, heat, salt, and UV-B stress. Integrated analysis with these data revealed a robust network: key miRNAs each targeted multiple circRNAs (**Figure 5A**) and numerous differentially expressed (DE) genes (**Figure 5B**).

Notably, the majority of these key hub miRNAs are cotton-specific, such as miR8633, miR8646, and miR8634, and strongly stress-responsive (**Figure 5C**), implying their evolved specializations in lineage-specific stress adaptation. Classifying DE circRNAs by their expression relative to host genes revealed a notable prevalence of “reversed” over “coordinated” patterns (**Figure 5D**), supporting that unlike canonical cleavage-based mRNA-miRNA interaction, these circRNAs may engage with miRNAs primarily through other modes. As a representative case, the miR8633 network under cold stress showed upregulation of all targeting circRNAs but downregulation of most targeted mRNAs (**Figure 5E**). This pattern is consistent with a model where circRNAs sponge miR8633, thereby alleviating its repression of target genes such as *Ga11g02685*—a putative homolog of *PRR5*, a negative regulator of cold tolerance in *Arabidopsis* (Guan et al., 2013). Similar miR8633-centered patterns under other stresses suggest this may be a recurrent regulatory module in *Gossypium* stress adaptation.

### 3.6 High abundance and locus specificity of chloroplast circRNAs in cotton

**Figure 6:**
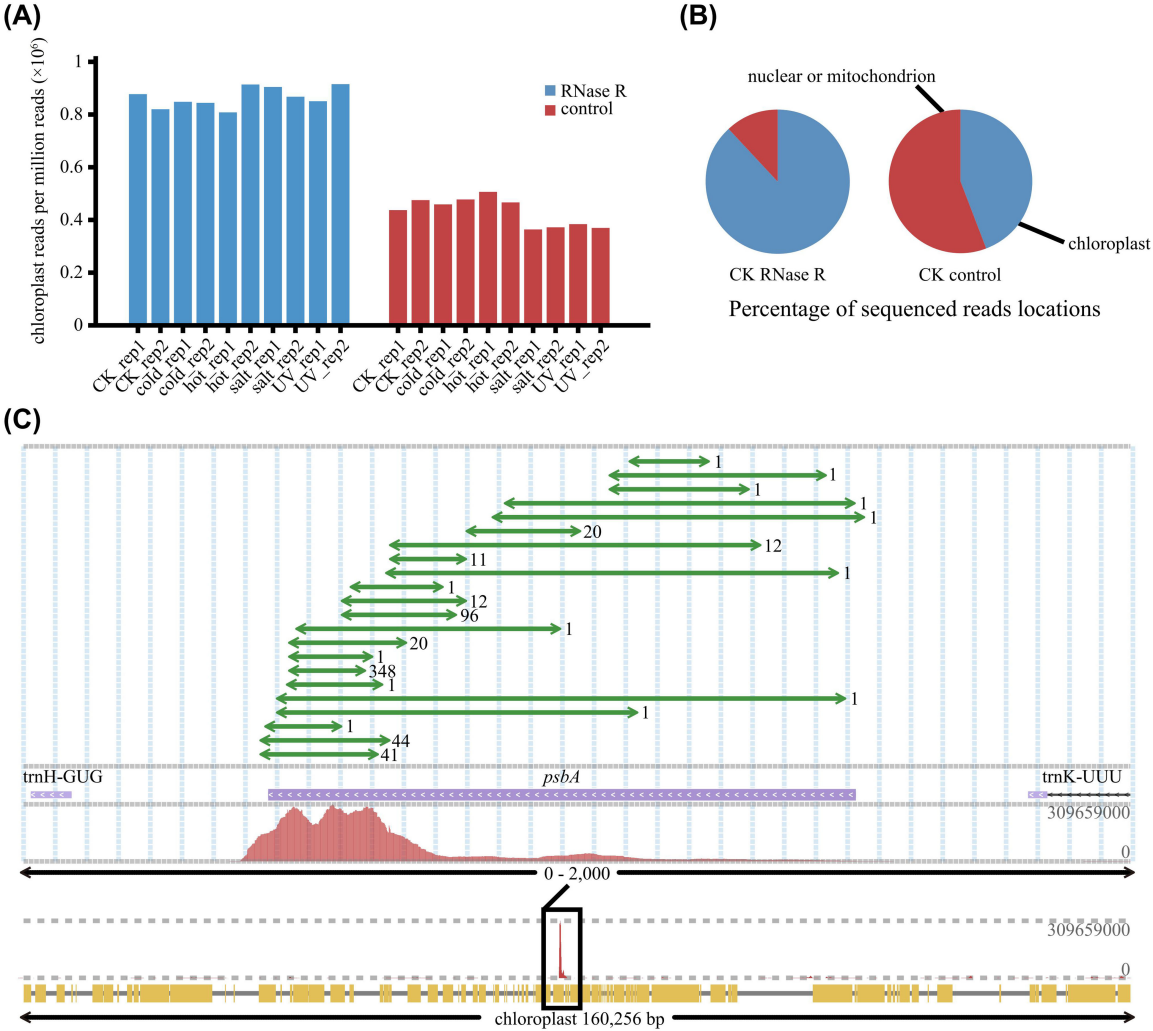
Characterization of chloroplast-derived circRNAs. **(A)** Comparison of the percentage of reads mapping to the chloroplast genome in circRNA-seq and RNA-seq libraries. **(B)** Pie charts illustrating the genomic distribution of sequenced reads. The left chart shows the proportion of reads mapping to the chloroplast genome versus the nuclear/mitochondrial genomes in the RNase R-treated sample, compared to the untreated control sample. **(C)** Genomic visualization of read coverage and BSJs at the chloroplast *psbA* locus. Arrows indicate the BSJ reads, verifying the circular structure at this locus.

Current research on plant circRNAs has primarily focused on those derived from nuclear genes via canonical back-splicing (Chen, 2020; Xiao et al., 2020). In contrast, our analysis in cotton revealed a remarkable enrichment of chloroplast-derived circRNAs, which constituted over 80% of the total circRNA pool (**Figures 6A, B**). This disproportionate abundance establishes the chloroplast as a hotspot of circRNA biogenesis and accumulation in cotton.

Mapping circRNA-seq reads across the chloroplast genome revealed a striking pattern: they were not randomly distributed but exclusively and specifically concentrated at the 3’ terminus of the core photosynthetic gene *psbA* (**Supplementary Table 6**). Furthermore, we detected multiple back-splicing isoforms from this locus, indicating complex alternative circularization (**Figure 6C**).

The *psbA* gene encodes the D1 protein, an essential component of the Photosystem II (PSII) reaction center. Due to its central role and susceptibility to photodamage, *psbA* expression is highly regulated (Nixon et al., 2010). The exceptional abundance and precise localization circRNAs at its 3’ terminus suggest a potential role in PSII homeostasis. We hypothesize that these chloroplast circRNAs may participate in the regulation of PSII repair and turnover, potentially by modulating *psbA* mRNA metabolism. This discovery unveils a novel, circRNA-associated layer of potential post-transcriptional regulation within the cotton chloroplast.

## 4 Discussion

### 4.1 Establishment of a high-confidence CircRNA landscape in *G. arboreum*

In this study, we established a high-resolution and high-confidence circRNA atlas for *G. arboreum* under multiple abiotic stresses. A significant challenge in plant circRNA research has been the high false-positive rate due to low abundance and interference from linear RNA degradation. We addressed this by employing an RNase R-enhanced RNA-seq strategy combined with a KNIFE-based algorithm pipeline. Our results demonstrate that RNase R treatment effectively depleted linear transcripts while enriching BSJs, significantly increasing the “Circ Fraction” across all stress treatments. This approach not only increased the number of identified circRNAs but also ensured high fidelity, as evidenced by our successful PCR and Sanger sequencing validation of 33 candidates. This comprehensive dataset significantly expands the known repertoire and understanding of circRNA in cotton.

### 4.2 Unique biogenesis mechanisms and evolutionary dynamics

The biogenesis of circRNAs in G. arboreum appears to diverge from the classical animal model. In animals, circRNA formation is primarily driven by Inverted Complementary Sequences (ICSs) in flanking introns (Zhang et al., 2014). However, our analysis revealed no significant enrichment of ICSs in cotton circRNAs. Instead, consistent with findings in other plants like Oryza sativa and Arabidopsis thaliana (Ye et al., 2015), we found that circRNA production is highly correlated with longer flanking introns, suggesting that plant-specific splicing factors or chromatin states might play a more dominant role. While the majority of identified circRNAs are derived from exonic back-splicing, the discovery of a significant portion of intronic circRNAs under abiotic stress suggests that stress-induced alternative splicing may trigger the circularization of non-canonical genomic regions, potentially serving as a rapid regulatory response to environmental fluctuations.

Our analysis revealed a pronounced decoupling between circRNA abundance and host gene expression, evidenced by a low median Pearson correlation coefficient of 0.34. This supports the “regulatory independence” model, suggesting that for the majority of the cotton circRNAome, accumulation is governed by autonomous biogenesis or stability control mechanisms rather than being a mere byproduct of host gene transcription. Such independence is an evolutionarily conserved strategy, aligning with large-scale transcriptomic observations in other major crops like rice and maize (Fan et al., 2025; Tang et al., 2018). Beyond this decoupling, we specifically identified “reversed patterns” where certain circRNAs are negatively correlated with the expression of their host genes. Such negative correlations (termed “reversed patterns” in our study) are common in both animals and plants, including poplar hybrids and maize (Abdelmohsen et al., 2017; Zhang et al., 2023; 2019). This aligns with the “competitive splicing” model, where circRNA biogenesis limits linear mRNA production by competing for splicing machinery or regulatory proteins (Wang et al., 2022). These findings reinforce the notion that circRNAs have evolved into independent functional entities, providing an additional layer of post-transcriptional control that contributes to the environmental resilience of cotton.

### 4.3 The dominance of organelle-derived circRNAs and psbA regulation

A notable finding of this study is the high abundance of chloroplast-derived circRNAs, which accounted for over 80% of total circRNA reads following RNase R treatment. Unlike their nuclear counterparts, these organellar molecules are specifically clustered at the 3’ terminus of the *psbA* gene. While nuclear circRNAs in cotton are associated with long flanking introns, chloroplast circRNAs likely stem from the high transcriptional flux of *psbA* and its unique 3’ terminus processing. Furthermore, the lack of 5’ caps and 3’ poly(A) tails in the chloroplast may inherently favor the formation and structural stability of covalently closed loops, protecting them from exonuclease-mediated degradation. As the structural and functional core of the Photosystem II (PSII) reaction center, *psbA* is indispensable for fine-tuning primary charge separation and ensuring photosynthetic resilience under fluctuating environmental conditions (Bhattacharjee et al., 2025). Given that its encoded D1 protein is highly susceptible to photoinhibition and requires rapid turnover (Aro et al., 1993), the striking enrichment of circRNAs at this pivotal locus suggests a specialized layer of post-transcriptional regulation. As *psbA* encodes the D1 protein, these circRNAs may serve as a conserved regulatory buffer to fine-tune photosynthetic machinery under stress. While the field has largely focused on nuclear-derived circRNAs (Li et al., 2018; Zhang et al., 2020), our results highlight the chloroplast—and specifically the *psbA* locus—as a critical yet underexplored hotspot for circRNA-mediated stress responses in cotton. The dominance of *psbA*-derived circRNAs in cotton likely arises from a synergy between high template availability and site-specific biogenesis. The preferential enrichment of 3’ terminus-derived reads following RNase R treatment (**Figure 6C**) supports an active circularization model, consistent with findings in *Arabidopsis* and other dicots where organelle circRNAs act as dynamic regulatory modules (Liu et al., 2019; Philips et al., 2020).

### 4.4 circRNAs in cross-stress tolerance

Our results demonstrate that a subset of circRNAs responds commonly to drought, cold, heat, and UV-B, suggesting their role in “cross-talk” pathways. One plausible mechanism, similar to that identified in cotton CMS-D2 restorer lines, is that they act as competitive endogenous RNAs (Wang et al., 2024). For instance, the enrichment of circRNAs originating from key stress-related loci identified in this study may allow them to modulate the plant’s core adaptive machinery by regulating their host transcripts or interacting with downstream signaling components. This aligns with the “rising star” role of plant circRNAs in fine-tuning cellular homeostasis under environmental pressure (Zhang and Dai, 2022).

Although their covalently closed loops generally provide high structural stability against exonucleases, their steady-state abundance represents a dynamic equilibrium between biogenesis and clearance (Chen, 2020; Xiao et al., 2020). In our study, the frequent decoupling of circRNA abundance from host gene levels (median r = 0.34) suggests that this equilibrium is independently and tightly controlled under abiotic stress. Even if some circRNAs undergo relatively rapid turnover, they may function as transient molecular switches or decoys to facilitate swift physiological adjustments to environmental fluctuations.

### 4.5 Perspectives and future directions

Despite the high-confidence atlas established here, several challenges remain. First, our identification relied on short-read sequencing, which has inherent limitations in fully resolving complex internal splicing patterns and full-length circRNA isoforms. To definitively establish the *in vivo* roles of specific candidates like *psbA*-derived circRNAs, future studies should integrate these emerging platforms with CRISPR/Cas9-based functional genomics, building upon the successful precedents established in rice (Zhou et al., 2021). Deciphering whether these molecules act as miRNA sponges or protein scaffolds will provide new avenues for molecular breeding of resilient cotton varieties in the face of global climate change. Deciphering whether these molecules act as miRNA sponges or protein scaffolds will provide new avenues for molecular breeding of resilient cotton varieties in the face of global climate change.

## Data Availability

The datasets presented in this study can be found in online repositories. The names of the repository/repositories and accession number(s) can be found in the article/**Supplementary Material**.

## Supplementary material

Supplementary Figure 1 Quality control and statistical analysis of RNA-seq data. **(A)** Bar chart showing the genomic distribution of mapped reads across all sequencing libraries. **(B)** Scatter plots evaluating the reproducibility of biological replicates. The x and y axes represent gene expression levels, and Pearson correlation coefficients are indicated for each comparison. **(C)** Venn diagram displaying the overlap of distinct back-splicing sites detected in RNase R-treated libraries versus standard RNA-seq libraries. **(D)** Pie charts illustrating the distribution of authenticity scores for back-splicing events identified in different datasets.

Supplementary Figure 2 Sanger-sequencing for the validation of candidate circRNAs. The chromatogram is shown under its corresponding nucleotide sequence. Red arrow represents the BSJs.

Supplementary Figure 3 Characterization of flanking sequences and evolutionary conservation of circRNAs. **(A)** Histogram comparing the length of the longest reverse complementary regions in the flanking sequences of circRNAs versus randomly selected exons. The *P*-value (0.62) indicates no significant difference, suggesting that flanking reverse repeats are not the primary drivers of circularization in this context. **(B)** Line plot showing the average repeat sequence coverage rate in the 3 kb flanking upstream and downstream regions of exonic circRNAs compared to random exons. **(C)** Profile of average repeat sequence coverage across the flanking introns of exonic circRNAs versus random introns. **(D)** Evolutionary conservation analysis of circRNA biogenesis. Gene models illustrate “Accordant”, “Half-accordant,” and “Unaccordant” circularization events between *G. arboreum* and orthologs in *A. thaliana*. The accompanying table summarizes the number of these events in *G. arboreum* versus *A. thaliana*, *O. sativa*, and *Z. mays*.

## References

[B1] Abdelmohsen K. Panda A. C. Munk R. Grammatikakis I. Dudekula D. B. De S. . (2017). Identification of HuR target circular RNAs uncovers suppression of PABPN1 translation by CircPABPN1. RNA Biol. 14, 361–369. doi: 10.1080/15476286.2017.1279788, PMID: 28080204 PMC5367248

[B2] Aro E.-M. Virgin I. Andersson B. (1993). Photoinhibition of Photosystem II. Inactivation, protein damage and turnover. Biochim. Biophys. Acta (BBA) - Bioenerg. 1143, 113–134. doi: 10.1016/0005-2728(93)90134-2, PMID: 8318516

[B3] Bhattacharjee S. Gordiy I. Sirohiwal A. Pantazis D. A. (2025). Microscopic basis of reaction center modulation in PsbA variants of photosystem II. Proc. Natl. Acad. Sci. 122, e2417963122. doi: 10.1073/pnas.2417963122, PMID: 40354529 PMC12107152

[B4] Chen L.-L. (2016). The biogenesis and emerging roles of circular RNAs. Nat. Rev. Mol. Cell Biol. 17, 205–211. doi: 10.1038/nrm.2015.32, PMID: 26908011

[B5] Chen L.-L. (2020). The expanding regulatory mechanisms and cellular functions of circular RNAs. Nat. Rev. Mol. Cell Biol. 21, 475–490. doi: 10.1038/s41580-020-0243-y, PMID: 32366901

[B6] Chen C. Li J. Feng J. Liu B. Feng L. Yu X. . (2021). sRNAanno—a database repository of uniformly annotated small RNAs in plants. Hortic. Res. 8, 45. doi: 10.1038/s41438-021-00480-8, PMID: 33642576 PMC7917102

[B7] Chen L. Zhang P. Fan Y. Lu Q. Li Q. Yan J. . (2018a). Circular RNAs mediated by transposons are associated with transcriptomic and phenotypic variation in maize. New Phytol. 217, 1292–1306. doi: 10.1111/nph.14901, PMID: 29155438

[B8] Chen S. Zhou Y. Chen Y. Gu J. (2018b). fastp: an ultra-fast all-in-one FASTQ preprocessor. Bioinformatics 34, i884–i890. doi: 10.1093/bioinformatics/bty560, PMID: 30423086 PMC6129281

[B9] Dai X. Zhao P. X. (2011). psRNATarget: a plant small RNA target analysis server. Nucleic Acids Res. 39, W155–W159. doi: 10.1093/nar/gkr319, PMID: 21622958 PMC3125753

[B10] Dobin A. Davis C. A. Schlesinger F. Drenkow J. Zaleski C. Jha S. . (2012). STAR: ultrafast universal RNA-seq aligner. Bioinformatics 29, 15–21. doi: 10.1093/bioinformatics/bts635, PMID: 23104886 PMC3530905

[B11] Du X. Huang G. He S. Yang Z. Sun G. Ma X. . (2018). Resequencing of 243 diploid cotton accessions based on an updated A genome identifies the genetic basis of key agronomic traits. Nat. Genet. 50, 796–802. doi: 10.1038/s41588-018-0116-x, PMID: 29736014

[B12] Enright A. J. John B. Gaul U. Tuschl T. Sander C. Marks D. S. (2003). MicroRNA targets in drosophila. Genome Biol. 5, R1. doi: 10.1186/gb-2003-5-1-r1, PMID: 14709173 PMC395733

[B13] Fahlgren N. Carrington J. C. (2010). miRNA target prediction in plants. Methods Mol. Biol. 592, 51–57. doi: 10.1007/978-1-60327-005-2_4, PMID: 19802588

[B14] Fan J. Zhang H. Zhou X. Gu L. Yao Y. Shi Y. . (2025). Genome-wide identification and characterization of circular RNAs involved in high night temperature stress at the filling stage of rice. Physiol. Plant 177, e70537. doi: 10.1111/ppl.70537, PMID: 40983998

[B15] Fan J. Quan W. Li G. B. Hu X. H. Wang Q. Wang H. . (2020). circRNAs Are Involved in the Rice-Magnaporthe oryzae Interaction. Plant Physiol. 2, 272–286. doi: 10.1104/pp.19.00716, PMID: 31628150 PMC6945833

[B16] Gao Z. Li J. Luo M. Li H. Chen Q. Wang L. . (2019). Characterization and cloning of grape circular RNAs identified the cold resistance-related vv-circATS1. Plant Physiol. 180, 966–985. doi: 10.1104/pp.18.01331, PMID: 30962290 PMC6548266

[B17] Guan Q. Wu J. Zhang Y. Jiang C. Liu R. Chai C. . (2013). A DEAD box RNA helicase is critical for pre-mRNA splicing, cold-responsive gene regulation, and cold tolerance in Arabidopsis. Plant Cell 25, 342–356. doi: 10.1105/tpc.112.108340, PMID: 23371945 PMC3584546

[B18] Hansen T. B. Jensen T. I. Clausen B. H. Bramsen J. B. Finsen B. Damgaard C. K. . (2013). Natural RNA circles function as efficient microRNA sponges. Nature 495, 384–388. doi: 10.1038/nature11993, PMID: 23446346

[B19] Jeck W. R. Sorrentino J. A. Wang K. Slevin M. K. Burd C. E. Liu J. . (2013). Circular RNAs are abundant, conserved, and associated with ALU repeats. Rna 19, 141–157. doi: 10.1261/rna.035667.112, PMID: 23249747 PMC3543092

[B20] Li H. Handsaker B. Wysoker A. Fennell T. Ruan J. Homer N. . (2009). The sequence alignment/map format and SAMtools. Bioinformatics 25, 2078–2079. doi: 10.1093/bioinformatics/btp352, PMID: 19505943 PMC2723002

[B21] Li N. Lin H. Wang T. Li Y. Liu Y. Chen X. . (2020). Impact of climate change on cotton growth and yields in Xinjiang, China. Field Crops Res. 247, 107590. doi: 10.1016/j.fcr.2019.107590, PMID: 41783259

[B22] Li X. Yang L. Chen L. L. (2018). The biogenesis, functions, and challenges of circular RNAs. Mol. Cell 71, 428–442. doi: 10.1016/j.molcel.2018.06.034, PMID: 30057200

[B23] Li Y. Zhu J. Xu J. Zhang X. Xie Z. Li Z. (2024). Effect of cold stress on photosynthetic physiological characteristics and molecular mechanism analysis in cold-resistant cotton (ZM36) seedlings. Front. Plant Sci. 15, 1396666. doi: 10.3389/fpls.2024.1396666, PMID: 38803600 PMC11128660

[B24] Liu S. Wang Q. Li X. Wang G. Wan Y. (2019). Detecting of chloroplast circular RNAs in Arabidopsis thaliana. Plant Signal Behav. 14, 1621088. doi: 10.1080/15592324.2019.1621088, PMID: 31130103 PMC6619943

[B25] Makarevitch I. Waters A. J. West P. T. Stitzer M. Hirsch C. N. Ross-Ibarra J. . (2015). Transposable elements contribute to activation of maize genes in response to abiotic stress. PLoS Genet. 11, e1004915. doi: 10.1371/journal.pgen.1004915, PMID: 25569788 PMC4287451

[B26] Ni Z. Liu N. Yu Y. Bi C. Chen Q. Qu Y. (2021). The cotton 70-kDa heat shock protein GhHSP70–26 plays a positive role in the drought stress response. Environ. Exp. Bot. 191, 104628. doi: 10.1016/j.envexpbot.2021.104628, PMID: 41783259

[B27] Nixon P. J. Michoux F. Yu J. Boehm M. Komenda J. (2010). Recent advances in understanding the assembly and repair of photosystem II. Ann. Bot. 106, 1–16. doi: 10.1093/aob/mcq059, PMID: 20338950 PMC2889791

[B28] OECD-FAO . (2024). OECD-FAO Agricultural Outlook 2024–2033 (Paris: OECD Publishing). Available online at: https://www.oecd.org/en/publications/oecd-fao-agricultural-outlook-2024-2033_4c5d2cfb-en.html (Accessed December 24, 2025).

[B29] Patil A. M. Pawar B. D. Wagh S. G. Shinde H. Shelake R. M. Markad N. R. . (2024). Abiotic stress in cotton: insights into plant responses and biotechnological solutions. Agriculture 14, 1638. doi: 10.3390/agriculture14091638, PMID: 41725453

[B30] Pertea M. Pertea G. M. Antonescu C. M. Chang T.-C. Mendell J. T. Salzberg S. L. (2015). StringTie enables improved reconstruction of a transcriptome from RNA-seq reads. Nat. Biotechnol. 33, 290–295. doi: 10.1038/nbt.3122, PMID: 25690850 PMC4643835

[B31] Philips A. Nowis K. Stelmaszczuk M. Jackowiak P. Podkowiński J. Handschuh L. . (2020). Expression Landscape of circRNAs in Arabidopsis thaliana Seedlings and Adult Tissues. Front. Plant Sci. 11, 576581. doi: 10.3389/fpls.2020.576581, PMID: 33014000 PMC7511659

[B32] Robinson M. D. Mccarthy D. J. Smyth G. K. (2009). edgeR: a Bioconductor package for differential expression analysis of digital gene expression data. Bioinformatics 26, 139–140. doi: 10.1093/bioinformatics/btp616, PMID: 19910308 PMC2796818

[B33] Sarwar M. Saleem M. F. Ullah N. Rizwan M. Ali S. Shahid M. R. . (2018). Exogenously applied growth regulators protect the cotton crop from heat-induced injury by modulating plant defense mechanism. Sci. Rep. 8, 17086. doi: 10.1038/s41598-018-35420-5, PMID: 30459328 PMC6244283

[B34] Song X. Yin X. Zhu Y. Su Q. Bao Y. (2024). Evolution of duplicated glutathione metabolic pathway in gossypium hirsutum and its response to UV-B stress. Ecol. Evol. 14, e70537. doi: 10.1002/ece3.70537, PMID: 39563703 PMC11575938

[B35] Su Y. Yi Y. Ge S. Wang Z. Wei Z. Liu X. . (2025). Circular RNAs derived from MIR156D promote rice heading by repressing transcription elongation of pri-miR156d through R-loop formation. Nat. Plants 11, 709–716. doi: 10.1038/s41477-025-01961-7, PMID: 40133670

[B36] Tang B. Hao Z. Zhu Y. Zhang H. Li G. (2018). Genome-wide identification and functional analysis of circRNAs in Zea mays. PLoS One 13, e0202375. doi: 10.1371/journal.pone.0202375, PMID: 30533052 PMC6289457

[B37] Textile Exchange . (2024). Materials Market Report 2024. Available online at: https://textileexchange.org/knowledge-center/reports/materials-market-report-2024/ (Accessed December 24, 2025).

[B38] Wang H. Gao X. Yu S. Wang W. Liu G. Jiang X. . (2022). Circular RNAs regulate parental gene expression: A new direction for molecular oncology research. Front. Oncol. 12, 947775. doi: 10.3389/fonc.2022.947775, PMID: 36091137 PMC9453195

[B39] Wang K. Wang D. Zheng X. Qin A. Zhou J. Guo B. . (2019a). Multi-strategic RNA-seq analysis reveals a high-resolution transcriptional landscape in cotton. Nat. Commun. 10, 4714. doi: 10.1038/s41467-019-12575-x, PMID: 31624240 PMC6797763

[B40] Wang Y. Xiong Z. Li Q. Sun Y. Jin J. Chen H. . (2019b). Circular RNA profiling of the rice photo-thermosensitive genic male sterile line Wuxiang S reveals circRNA involved in the fertility transition. BMC Plant Biol. 19, 340. doi: 10.1186/s12870-019-1944-2, PMID: 31382873 PMC6683460

[B41] Wang R. Zhang M. Wang H. Chen L. Zhang X. Guo L. . (2024). Identification and characterization of circular RNAs involved in the fertility stability of cotton CMS-D2 restorer line under heat stress. BMC Plant Biol. 24, 32. doi: 10.1186/s12870-023-04706-w, PMID: 38183049 PMC10768462

[B42] Wang G. Zhang Q. Woolway R. I. Xu L. Ma H. Yang Z. (2025). Warm-wetting and/or warm-drying tendency over Xinjiang, China? J. Hydrol. 660, 133417. doi: 10.1016/j.jhydrol.2025.133417, PMID: 41783259

[B43] Wu Y. Liu F. Yang D. G. Li W. Zhou X. J. Pei X. Y. . (2018). Comparative chloroplast genomics of gossypium species: insights into repeat sequence variations and phylogeny. Front. Plant Sci. 9, 376. doi: 10.3389/fpls.2018.00376, PMID: 29619041 PMC5871733

[B44] Xiao M.-S. Ai Y. Wilusz J. E. (2020). Biogenesis and functions of circular RNAs come into focus. Trends Cell Biol. 30, 226–240. doi: 10.1016/j.tcb.2019.12.004, PMID: 31973951 PMC7069689

[B45] Xu Y. Tian S. Cong X. Bai M. Zhang J. (2025). Projected hydroclimatic changes in Xinjiang under bias-corrected CMIP6 scenarios. Front. Plant Sci. 16, 1679735. doi: 10.3389/fpls.2025.1679735, PMID: 41446676 PMC12722945

[B46] Yao J. Mao W. Chen J. Dilinuer T. (2021). Recent signal and impact of wet-to-dry climatic shift in Xinjiang, China. J. Geogr. Sci. 31, 1283–1298. doi: 10.1007/s11442-021-1898-9, PMID: 41784122

[B47] Ye C.-Y. Chen L. Liu C. Zhu Q.-H. Fan L. (2015). Widespread noncoding circular RNAs in plants. New Phytol. 208, 88–95. doi: 10.1111/nph.13585, PMID: 26204923

[B48] Yin Z. Zhao Q. Lv X. Zhang X. Wu Y. (2024). Circular RNA ath-circ032768, a competing endogenous RNA, response the drought stress by targeting miR472-RPS5 module. Plant Biol. (Stuttg) 26, 544–559. doi: 10.1111/plb.13645, PMID: 38588338

[B49] Zafar M. M. Chattha W. S. Khan A. I. Zafar S. Subhan M. Saleem H. . (2023). Drought and heat stress on cotton genotypes suggested agro-physiological and biochemical features for climate resilience. Front. Plant Sci. 14, 1265700. doi: 10.3389/fpls.2023.1265700, PMID: 38023925 PMC10643170

[B50] Zhang P. Dai M. (2022). CircRNA: a rising star in plant biology. J. Genet. Genomics 49, 1081–1092. doi: 10.1016/j.jgg.2022.05.004, PMID: 35644325

[B51] Zhang P. Fan Y. Sun X. Chen L. Terzaghi W. Bucher E. . (2019). A large-scale circular RNA profiling reveals universal molecular mechanisms responsive to drought stress in maize and Arabidopsis. Plant J. 98, 697–713. doi: 10.1111/tpj.14267, PMID: 30715761

[B52] Zhang P. Li S. Chen M. (2020). Characterization and function of circular RNAs in plants. Front. Mol. Biosci. 7, 91. doi: 10.3389/fmolb.2020.00091, PMID: 32509801 PMC7248317

[B53] Zhang H. Li D. Zhou Z. Zahoor R. Chen B. Meng Y. (2017). Soil water and salt affect cotton (Gossypium hirsutum L.) photosynthesis, yield and fiber quality in coastal saline soil. Agric. Water Manage. 187, 112–121. doi: 10.1016/j.agwat.2017.03.019, PMID: 41783259

[B54] Zhang X. O. Wang H. B. Zhang Y. Lu X. Chen L. L. Yang L. (2014). Complementary sequence-mediated exon circularization. Cell 159, 134–147. doi: 10.1016/j.cell.2014.09.001, PMID: 25242744

[B55] Zhang W. Yuan Z. Zhang J. Su X. Huang Q. Liu Q. . (2023). Identification and functional prediction of circRNAs in leaves of F1 hybrid poplars with different growth potential and their parents. Int. J. Mol. Sci. 24, 2284. doi: 10.3390/ijms24032284, PMID: 36768607 PMC9916877

[B56] Zheng X. Chen Y. Zhou Y. Shi K. Hu X. Li D. . (2020). Full-length annotation with multistrategy RNA-seq uncovers transcriptional regulation of lncRNAs in cotton. Plant Physiol. 185, 179–195. doi: 10.1093/plphys/kiaa003, PMID: 33631798 PMC8133545

[B57] Zhou J. Yuan M. Zhao Y. Quan Q. Yu D. Yang H. . (2021). Efficient deletion of multiple circle RNA loci by CRISPR-Cas9 reveals Os06circ02797 as a putative sponge for OsMIR408 in rice. Plant Biotechnol. J. 19, 1240–1252. doi: 10.1111/pbi.13544, PMID: 33440058 PMC8196656

