# Supplementary figures and images for: A comprehensive landscape of the Gossypium arboreum circRNAome under multiple abiotic stresses
Source: Front Plant Sci. 2026 Mar 10;17:1791897. doi: 10.3389/fpls.2026.1791897 (PMC13008959; doi:10.3389/fpls.2026.1791897)

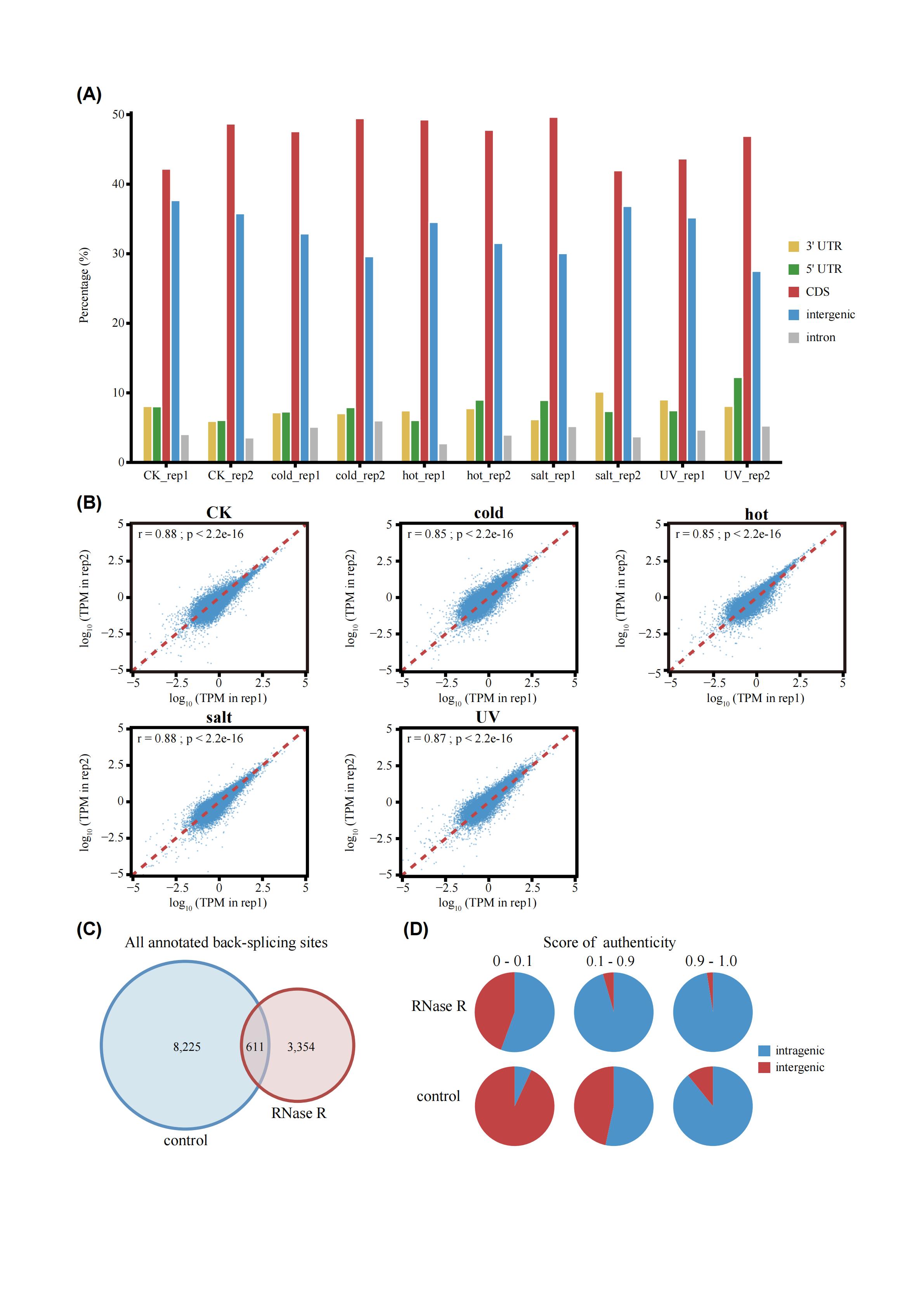

Supplement: Supplementary Figure 1 — Quality control and statistical analysis of RNA-seq data. (A) Bar chart showing the genomic distribution of mapped reads across all sequencing libraries. (B) Scatter plots evaluating the reproducibility of biological replicates. The x and y axes represent gene expression levels, and Pearson correlation coefficients are indicated for each comparison. (C) Venn diagram displaying the overlap of distinct back-splicing sites detected in RNase R-treated libraries versus standard RNA-seq libraries. (D) Pie charts illustrating the distribution of authenticity scores for back-splicing events identified in different datasets. [file Image1.jpeg]

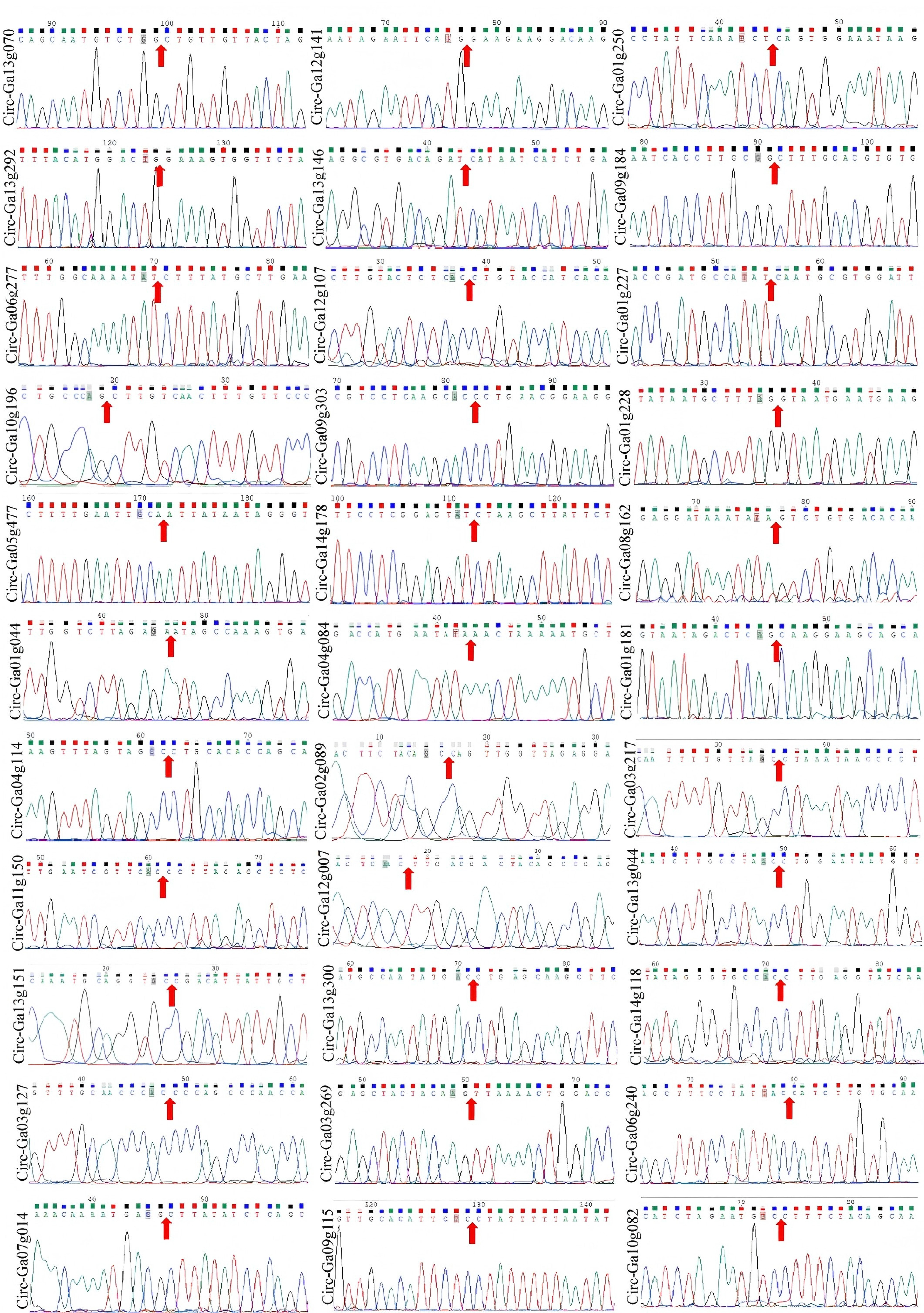

Supplement: Supplementary Figure 2 — Sanger-sequencing for the validation of candidate circRNAs. The chromatogram is shown under its corresponding nucleotide sequence. Red arrow represents the BSJs. [file Image2.jpeg]

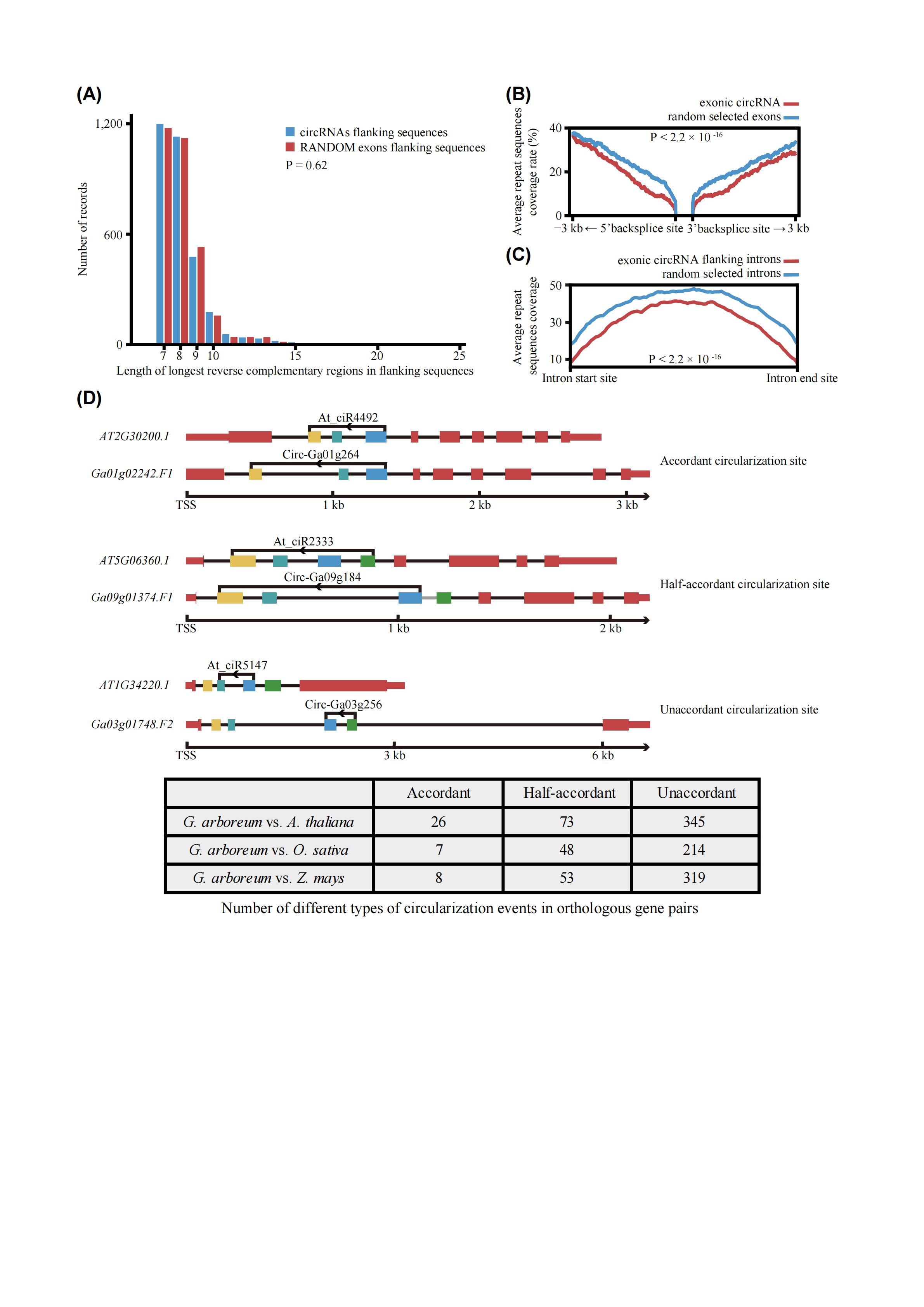

Supplement: Supplementary Figure 3 — Characterization of flanking sequences and evolutionary conservation of circRNAs. (A) Histogram comparing the length of the longest reverse complementary regions in the flanking sequences of circRNAs versus randomly selected exons. The P-value (0.62) indicates no significant difference, suggesting that flanking reverse repeats are not the primary drivers of circularization in this context. (B) Line plot showing the average repeat sequence coverage rate in the 3 kb flanking upstream and downstream regions of exonic circRNAs compared to random exons. (C) Profile of average repeat sequence coverage across the flanking introns of exonic circRNAs versus random introns. (D) Evolutionary conservation analysis of circRNA biogenesis. Gene models illustrate “Accordant”, “Half-accordant,” and “Unaccordant” circularization events between G. arboreum and orthologs in A. thaliana. The accompanying table summarizes the number of these events in G. arboreum versus A. thaliana, O. sativa, and Z. mays. [file Image3.jpeg]
